# Pneumocephalus secondary to epidural analgesia: a case report

**DOI:** 10.1186/s13256-023-03955-5

**Published:** 2023-05-26

**Authors:** Maira Ahmad, Shannay Bellamy, William Ott, Rany Mekhail

**Affiliations:** Jersey City Medical Center, Jersey, NJ 07302 USA

**Keywords:** Pneumocephalus, Epidural anesthesia, Post-dural puncture headache, Headache

## Abstract

**Introduction:**

Epidural anesthesia is commonly used for analgesia during labor, and headache is a common complaint following this procedure. Pneumocephalus, on the other hand, is a rare and potentially serious complication of epidural anesthesia, which is most often caused by accidental puncture of the dura with the introduction of air into intrathecal space.

**Case presentation:**

We present the case of a 19-year-old Hispanic female who developed a severe frontal headache and neck pain eight hours following epidural catheter placement to deliver analgesia during labor. Physical examination was within normal limits without any neurological deficits. Computed tomography of the head and neck would later demonstrate small to moderate amounts of pneumocephalus, predominantly within the frontal horn of the lateral ventricles, and a moderate amount of air within the spinal canal. She was treated conservatively with analgesia. Though headache recurred after discharge, repeat imaging showed improvement in the volume of pneumocephalus and conservative management was continued.

**Conclusions:**

Although a rare complication and an uncommon cause of headache following epidural anesthesia, a high index of suspicion must remain for pneumocephalus as it may cause significant morbidity and, in some cases, be potentially life-threatening.

## Introduction

Epidural anesthesia is commonly used for analgesia during labor, and headache is a common complaint following this procedure. In most cases, headache following epidural puncture is due to post-dural puncture headache (PDPH) characterized by cerebrospinal fluid leakage from the dural puncture resulting in low intracranial pressure [1]. Patients with PDPH often present with occipital or frontal headaches worsened in the upright position and may be associated with tinnitus, visual changes, nausea, and vomiting. PDPH is often self-limiting and, in most instances, resolves without interventions [2]. Pneumocephalus, on the other hand, is a rare and potentially serious complication of epidural anesthesia, which is most often caused by accidental puncture of the dura with the introduction of air into intrathecal space [3]. Patients with pneumocephalus present with postural headaches, which may be quite similar in character to PDPH therefore, a thorough history and high level of suspicion are needed to make the diagnosis. Here we present the case of a 19-year-old female who developed pneumocephalus after epidural catheter placement to deliver analgesia during labor.

## Case presentation

19-year-old primigravida Hispanic female with no past medical history or family history presented to the antenatal unit at 40 weeks and one day for elective induction of labor. She had an uncomplicated pregnancy, had regular antenatal follow-up visits, and was not taking any medications. On presentation, she reported no complaints. Vital signs were all within normal limits and the physical exam was significant for a gravid uterus with a size corresponding to her gestational age. Labor was induced with misoprostol followed by oxytocin infusion.

For the management of labor pain when she had reached about 3 cm of cervical dilatation, epidural analgesia was requested. Epidural puncture was performed in the L4-L5 intervertebral space with the patient in the sitting position. The patient was monitored during the procedure with non-invasive blood pressure monitoring, pulse oximetry and cardiac monitoring. The procedure was noted to be technically difficult with a noted tachycardia during the procedure, which resolved spontaneously. After successful placement, she was commenced on analgesia with Fentanyl as needed. 8 h following epidural catheter placement, she complained of severe frontal headache and neck pain. Headache and neck pain were described as 10/10 in severity, pounding in nature, non-radiating and worsened with positioning and movement of the head and neck. She denied any visual disturbances, photophobia, nausea or limb weakness. Vitals had remained within normal limits and a neurological examination at that time was unremarkable. At that time, differential diagnosis for her new onset headache and neck pain post-difficult epidural puncture included subarachnoid puncture with acute loss of cerebrospinal fluid (CSF), post-dural puncture headache and pneumocephalus. Though she was experiencing headaches and neck pain, the epidural analgesia was noted to be effective for the management of labor pain and was continued. She was observed closely and reviewed frequently, during which time she had reported improvement in the severity of the headache.

Due to failure to progress in the first stage of pregnancy, she was taken to the Cesarean section without complications. She still complained of a persistent headache following the Cesarean section but reported that the headache had improved.

**Fig. 1:**
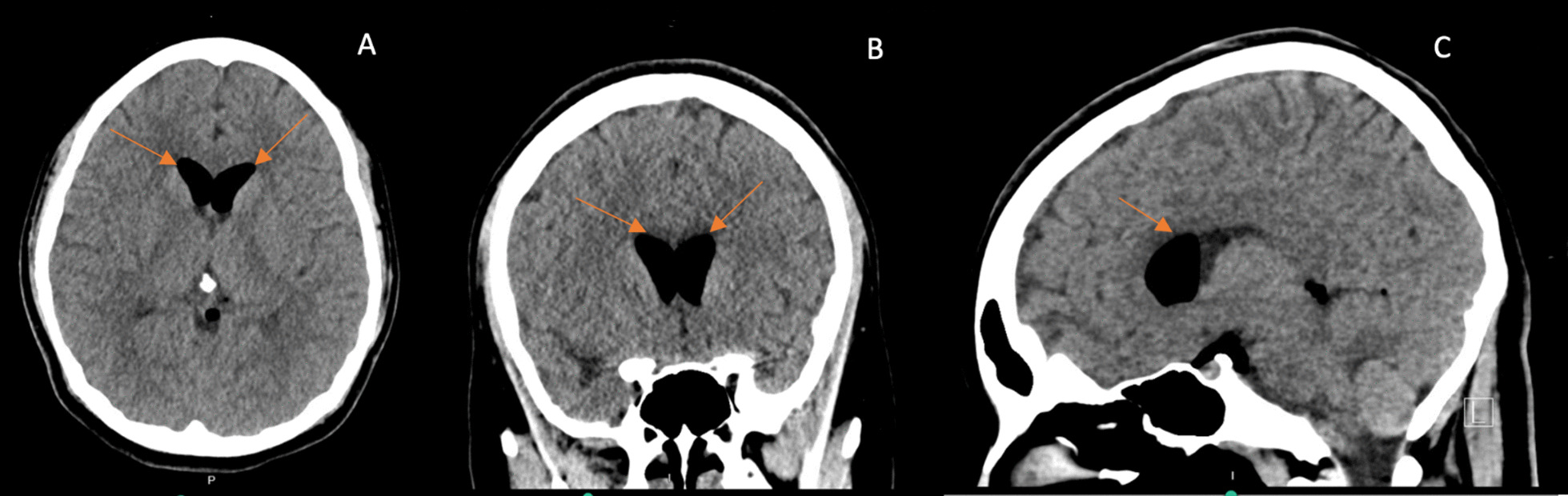
Computed tomography head showing air predominantly within the frontal horn of the lateral ventricles without midline shift or mass effect. **A** Axial view, **B** Coronal view, **C** Sagittal view. Orange arrows identify the abnormal presence of air

**Fig. 2:**
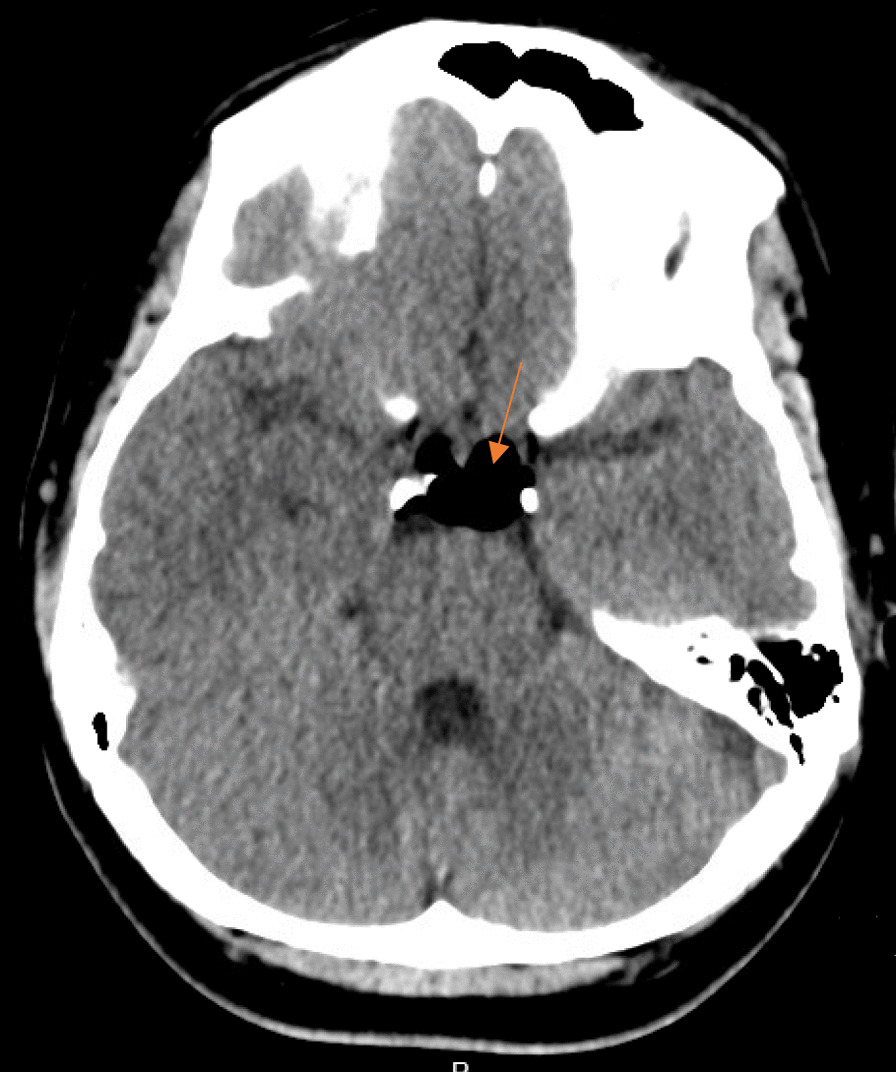
Computed tomography head showing air within the suprasellar cistern. Orange arrows identify the abnormal presence of air

**Fig. 3:**
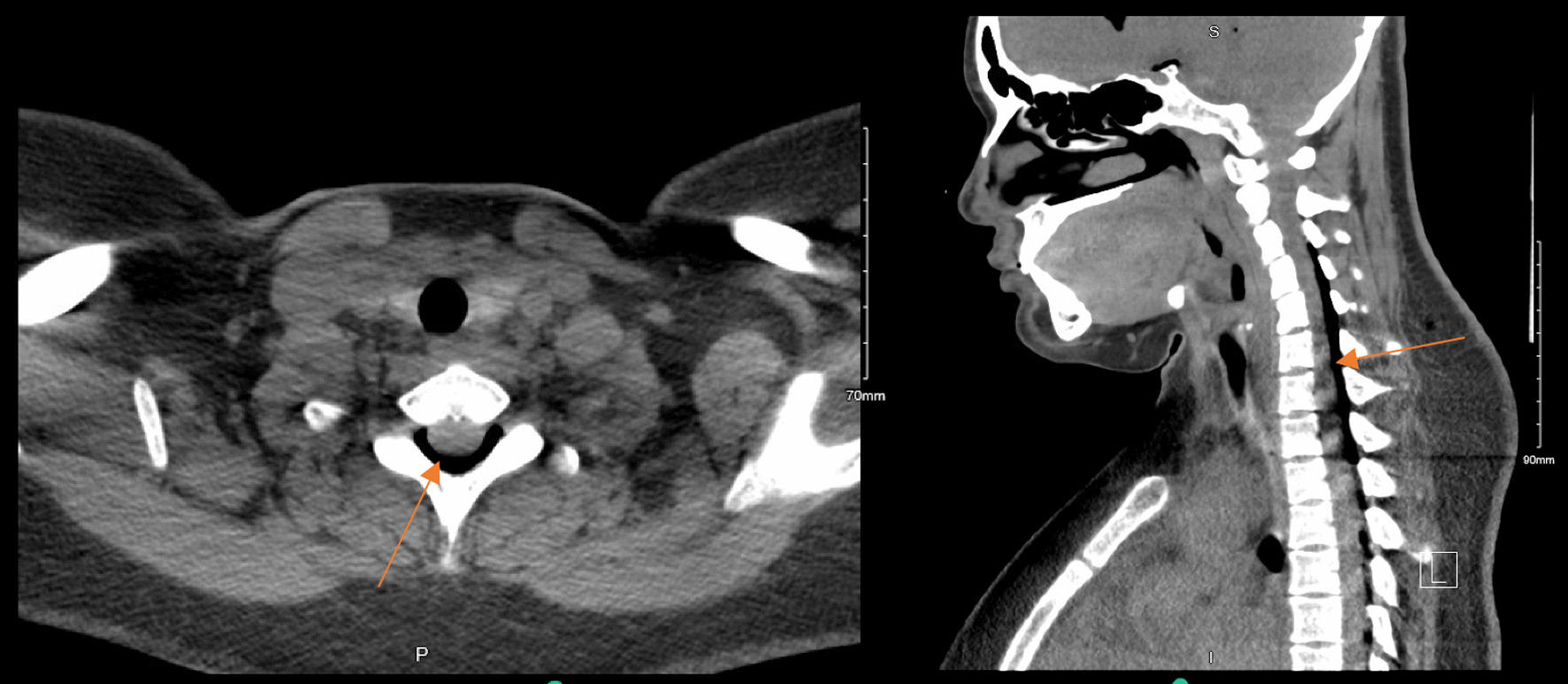
Computed tomography neck showing air within the cervical spinal canal. **A** Axial view, **B** Sagittal view. Orange arrows identify the abnormal presence of air

On postoperative day (POD) 1, a computed tomography (CT) of the head and neck was done on the advice of the anesthetic team, as the patient was still complaining of persistent positional headaches, which had been waxing and waning in intensity. CT head was significant for a small to moderate amount of pneumocephalus predominantly within the frontal horn of the lateral ventricles and, to a lesser extent, the suprasellar cistern and perimesencephalic cisterns without significant mass effect, midline shift or hydrocephalus (Figs. 1 and 2). There was no noted intracerebral hemorrhage or infarct seen. The CT neck showed moderate air within the spinal canal predominantly within the dorsal epidural space—throughout the cervical spine and visualized in the upper thoracic spine (Fig. 3). She was followed up regularly by the anesthetic team, with close monitoring and conservative management with analgesia as needed.

On POD 2 she reported feeling much better with improvement of the headache. She was ambulating without difficulty and without any worsening of headache and neck pain. She was subsequently discharged on POD 3 with recommendations to return for worsening headache or neck pain, vision changes, limb weakness, heavy vaginal bleeding, fever, or foul-smelling vaginal discharge.

**Fig. 4:**
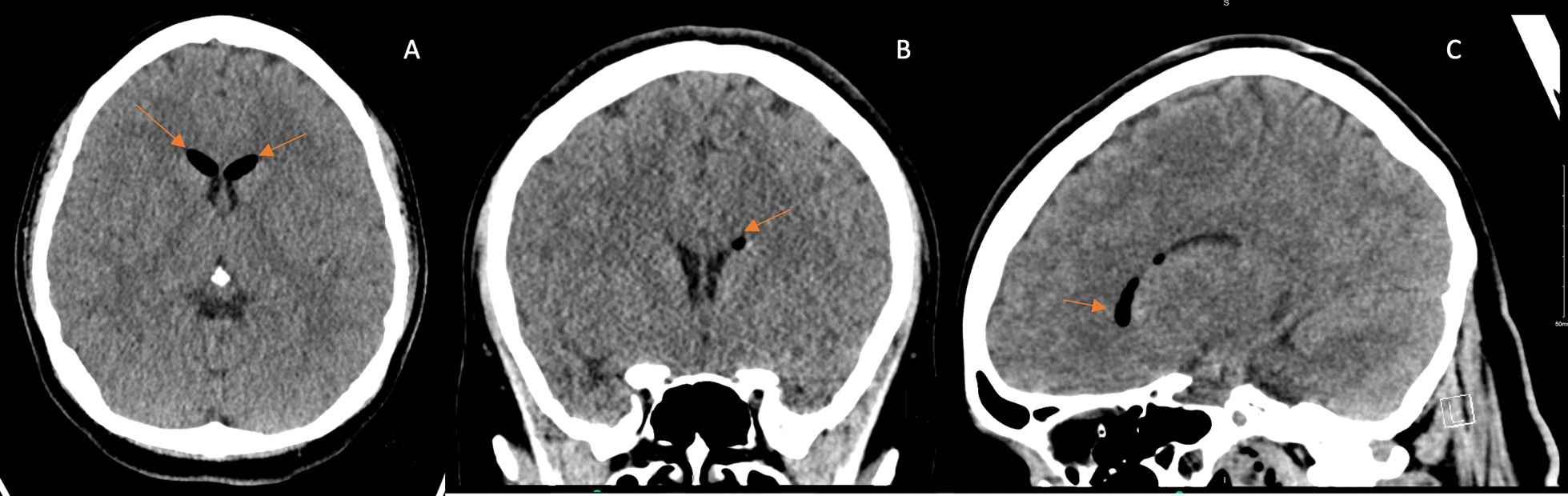
Computed tomography Head on the second presentation showing improvement in the intracranial air. **A** Axial view, **B** Coronal view, **C** Sagittal view. Orange arrows identify the abnormal presence of air

**Fig. 5:**
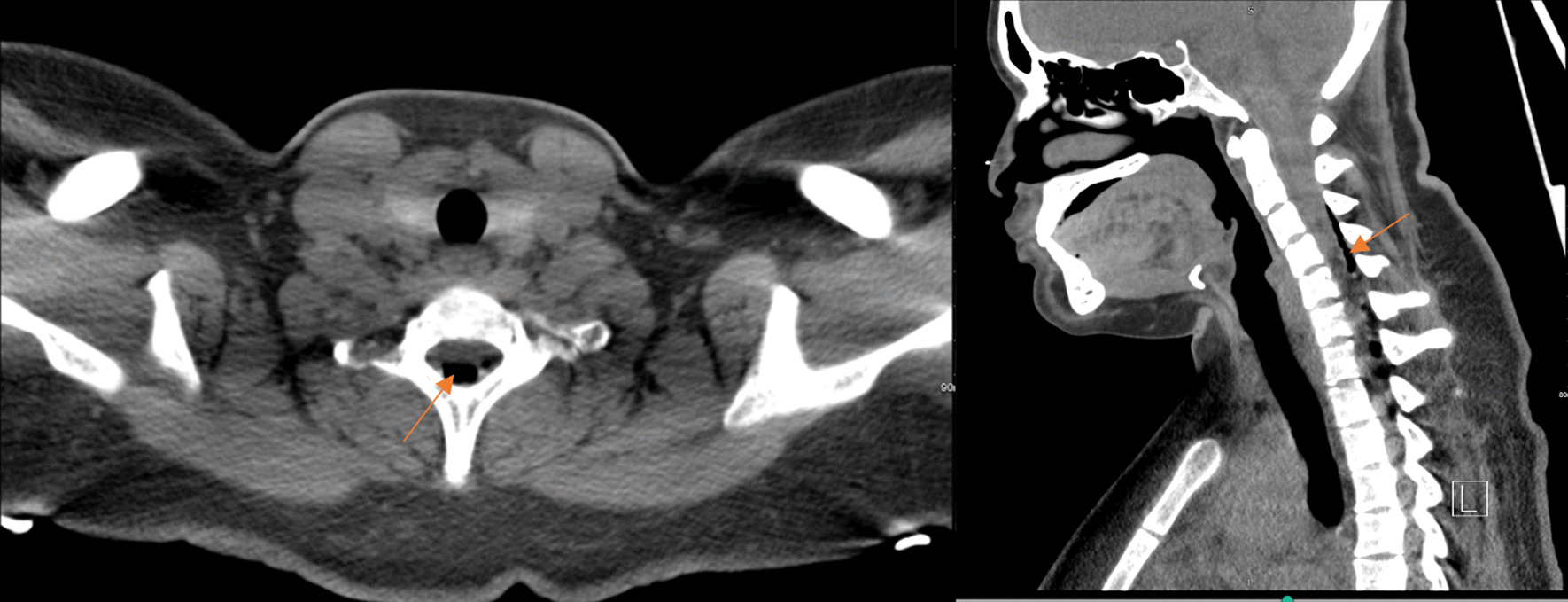
Computed tomography of the neck on the second presentation showing decreased small dorsal cervical/upper thoracic spine epidural air. **A** Axial view, **B** Sagittal view. Orange arrows identify the abnormal presence of air

She initially reported good relief of headaches with analgesia. However, two days following discharge, she presented with frontal headaches that gradually worsened to 10/10 severity despite analgesia with acetaminophen and oxycodone. She described the pain as radiating to her neck and bilateral arms with numbness and paresthesias to the arms bilaterally. On this presentation, she was hemodynamically stable and afebrile with normal oxygen saturations on room air. Her physical exam was significant for a well-opposed Cesarean scar without bleeding or discharge and revealed no focal motor or sensory, neurological deficits. Repeat CT head imaging at that time showed decreased small intraventricular and left perimesencephalic cistern pneumocephalus without any hydrocephalus, bleed or infarct and repeat CT neck showed decreased small dorsal cervical/upper thoracic spine epidural air when compared to the previous scan (Figs. 4 and 5). Anesthesia and Neurosurgery were consulted. She was treated with conservative management with analgesia (this time with a combination of butalbital, acetaminophen, and caffeine), aggressive intravenous fluid hydration, rest, and caffeine during the day while active. A blood patch was not considered initially, as there were concerns that increasing pressure within the epidural space would further worsen her symptoms. Over the following two days, she gradually resolved her headaches and neck pain and was subsequently discharged home.

## Discussion

Pneumocepahlus—otherwise sometimes referred to as pneumatocele or intracranial aerocele—is defined as the presence of air in the intracranial space. Over the years, it has typically been found to occur following trauma, cranial surgeries, and infections [4]. Pneumocephalus is an infrequent complication of a spinal or epidural puncture due to an accidental dural puncture, allowing air entry into the subdural or subarachnoid space [5]. The incidence of an accidental dural puncture during epidural anesthesia is about 0.06%. However, the true incidence of pneumocephalus is very difficult to establish, mainly due to the fact that available literature largely reflects a few cases reported annually [6].

Several mechanisms have been proposed over the years regarding the pathophysiology for the development of pneumocephalus secondary to accidental dural puncture. One of the more recognized theories is the “ball-valve” theory first described by Dandy in 1926. This theory proposed that air may enter the cranium through a dural membrane defect caused by an accidental dural puncture, which acts as a one-way valve—the dural defect allows air to enter the dural space, but subsequently closes, preventing any outflow of air. It is thought that the prevention of air outflow is caused by an increase in intracranial pressure (ICP) that forces the brain parenchyma to block the entry site [7]. Another proposed mechanism is the “inverted soda bottle effect,” or vacuum effect, first established by Horowitz in 1964. It suggests that the cerebrospinal fluid (CSF) leakage caused by a dural puncture may lead to that negative ICP which may pull air into the intracranial space. The air replaces the lost fluid volume via a pressure gradient between the extracranial and intracranial compartments [7–9].

Pneumocephalus can be classified based on the timing of either acute pneumocephalus—occurring within less than 72 h of dural puncture or delayed pneumocephalus which occurs more than 72 h following dural puncture [10]. The severity of pneumocephalus can be characterized into two types—simple pneumocephalus and tension pneumocephalus. Simple pneumocephalus is a benign accumulation of air in the intracranial cavity without any significant increase in intracranial pressure, compression of brain parenchyma, or brain herniation. It is most commonly a complication of neurosurgical procedures. On the other hand, tension pneumocephalus is a life-threatening condition in which intracranial accumulation of air leads to a significant increase in transcranial pressure with associated brain herniation [11]. Most cases of pneumocephalus following epidural puncture are often characterized as simple pneumocephalus.

Headache is the most common presenting symptom of pneumocephalus. It is often localized to the frontal or occipital regions and postural in nature, worsening in the upright position similar in presentation to PDPH, a more common cause of headache following epidural or spinal punctures. Similarly, both PDPH and pneumocephalus may be associated with nausea, vomiting, neck pain or stiffness, changes in vision, and cranial nerve deficit. Therefore, the timeline of the events and a high level of clinical suspicion are needed to make the diagnosis [3]. Unlike PDPH, which can occur gradually over 24–28 h following dural puncture, the headache of pneumocephalus is often sudden in onset and severe. It may be worsened not only with upright positioning but with general movement and often may not resolve in the recumbent position [3].

Our patient presented with sudden onset severe bifrontal headache and neck, worsening by movement, eight hours following difficult epidural analgesia for pain management during labor. Given the difficulty of the procedure, an accidental dural puncture was considered, with PDPH initially considered the primary differential diagnosis. Given the persistent nature of the headache, a CT of the head was done after an emergent Cesarean section, which revealed air within the intracranial space and in the cervical spinal canal without any evidence of mass effect or herniation—simple pneumocephalus.

A non-contrast head CT is the most common test for diagnosing pneumocephalus due to its high sensitivity and specificity, even for identifying minimal air. It can be helpful in differentiating the more benign simple pneumocephalus from tension pneumocephalus [3, 5].

Following diagnosis, the treatment of pneumocephalus often depends on the severity. In most cases, simple pneumocephalus resolves without intervention as the intracranial air may be absorbed over the following two to three weeks. Conservative management of simple pneumocephalus with simple analgesia is the delivery of oxygen at a concentration of 40–100% in the supine or recumbent position to increase the intracranial air absorption rate [9]. On the other hand, tension pneumocephalus is a neurosurgical emergency. It requires prompt surgical intervention by aspiration of the air by needle decompression, burr hole placement, or alternatively closure of the dural defect leading to air accumulation [9, 12].

Our patient’s simple pneumocephalus was managed conservatively with simple analgesia, though she did experience a recurrence of her headache following initial improvement and discharge. However, at the time she represented, CT imaging showed significant improvement of the intracranial air suggesting that her pneumocephalus was resolving. Therefore, conservative management continued.

## Conclusion

In conclusion, pneumocephalus remains an extremely rare complication of spinal or epidural-related procedures worldwide. Careful attention must be exhibited not only during the procedure in question itself but also during the time thereafter in which symptoms of complication present. While most cases can be managed conservatively, all cases ought to be worked up thoroughly in the event a tension pneumocephalus is in play and emergent neurosurgical intervention becomes warranted. Further research and data pooling is needed to characterize how frequently this pathology occurs, how different patients present, and if new interventional techniques can be developed—so that patient care can continue to become as efficient and safe as possible.

## Data Availability

Not applicable.

## References

[1] Anwari JS, Hazazi AA (2015). Another cause of headache after epidural injection. Neurosciences.

[2] Yates H, Hamill M, Borel CO, Toung TJ (1994). Incidence and perioperative management of tension pneumocephalus following craniofacial resection. J Neurosurg Anesthesiol.

[3] Reddi S, Honchar V, Robbins MS (2015). Pneumocephalus associated with epidural and spinal anesthesia for labor. Neurol Clin Pract.

[4] Jelsma F, Moore DF (1954). Cranial aerocele. Am J Surg.

[5] Nistal-Nuño B, Gómez-Ríos MÁ (2014). Case report: pneumocephalus after labor epidural anesthesia. Res.

[6] Hawley JS, Ney JP, Swanberg MM (2005). Subarachnoid pneumocephalus from epidural steroid injection. Headache.

[7] Vacca VM (2017). Pneumocephalus assessment and management. Nurs Crit Care.

[8] Wang A, Solli E, Carberry N, Hillard V, Tandon A (2016). Delayed tension pneumocephalus following gunshot wound to the head: a case report and review of the literature. Case Rep Surg.

[9] Dabdoub CB, Salas G, Silveira Edo N, Dabdoub CF (2015). Review of the management of pneumocephalus. Surg Neurol Int.

[10] Pillai P, Sharma R, MacKenzie L, Reilly EF, Beery PR, Papadimos TJ, Stawicki SP (2017). Traumatic tension pneumocephalus—two cases and comprehensive review of literature. Int J Crit Illn Inj Sci.

[11] Wankhade BS, Beniamein MMK, Alrais ZF, Mathew JI, Alrais GZ. What should an intensivist know about pneumocephalus and tension pneumocephalus? In: Acute and critical care. The Korean Society of Critical Care Medicine. 2022. 10.4266/acc.2021.01102 10.4266/acc.2021.01102 PMC10265419 35545242

[12] Haj R, Devanabanda B, Louis M (2020). Iatrogenic pneumocephalus complicated by cardiac arrest in a peripartum patient. Consultant.

